# Indian Spring

**Published:** 1874-08

**Authors:** 


We have received a pamphlet giving a condensed history of the Indian Spring in Butts county, Georgia, with chemical analysis and account of its medicinal virtues. The reputation of this long established watering place has stood the test of time and is fully maintained. Ample provision is made for the comfortable entertainment of visitors.

One of our Subscribers upon the Chattahoochee, in the cotton belt, puts it strongly: "The practice of medicine does not pay here now as formerly. The people are greatly impoverished. The luxuries of whisky, tobacco and coffee seem indispensable to them, and if they have any cash left from the purchase of these, it goes for the ever present *patent medicine* to be found in all our country stores. The regular physician must now practice for an unlimited credit, and lay up for himself treasure where neither moth nor rust doth corrupt, for gold and silver hath he none."

